# Forms of Health System Fragmentation During Conflict: The Case of Yemen

**DOI:** 10.3389/fpubh.2021.659980

**Published:** 2021-07-12

**Authors:** Fekri Dureab, Taha Hussain, Rashad Sheikh, Najwa Al-Dheeb, Sameh Al-Awlaqi, Albrecht Jahn

**Affiliations:** ^1^Heidelberg Institute of Global Health, Heidelberg University, Heidelberg, Germany; ^2^Institute for Research in International Assistance, Akkon Hochschule, Berlin, Germany; ^3^MSF-France, Aden, Yemen; ^4^Health Systems, Policy and Management Expert, Sana'a, Yemen; ^5^Public Health Expert, Sana'a, Yemen; ^6^Yemeni Public Health Expert, Berlin, Germany

**Keywords:** health system, fragmentation forms, Yemen, UHC, conflict, qualitative study

## Abstract

The continuous and protracted conflict in Yemen has evolved into the worst humanitarian situation in modern history. All public structures in the country, including the health system and its basic functions, have been under tremendous pressures. One of the key obstacles to improve the health outcomes in Yemen is fragmentation of the health system. This study aims at exploring and documenting the forms of health system fragmentation in humanitarian and conflict-affected contexts by studying Yemen as a case study. We collected national qualitative data from key informants through in-depth interviews. A pool of respondents was identified from the Ministry of Public Health and Population, donors, and non-governmental organizations. Data were collected between May and June 2019. We interviewed eight key informants and reviewed national health policy documents, and references provided by key informants. Interviews were recorded, transcribed, and analyzed using qualitative content analysis. We further conducted a literature review to augment and triangulate the findings. Six themes emerged from our datasets and analyses, representing various forms of fragmentation: political, structural, inter-sectoral, financial, governance, and health agenda-related forms. Health system fragmentation in Yemen existed before the conflict eruption and has aggravated as the conflict evolves. The humanitarian situation and the collapsing health system enabled the influx of various national and international health actors. In conclusion, the protracted conflict and fragile situation in Yemen have accentuated the fragmentation of the health system. Addressing these fragmentations' forms by all health actors and building consensus on health system agenda are recommended. Health system analysis and in-depth study of fragmentation drivers in Yemen can be beneficial to build common ground and priorities to reduce health system fragmentation. Furthermore, capacity building of a health system is fundamental for the humanitarian development nexus, health system integration, and recovery efforts in the future.

## Introduction

Health system fragmentation occurs when there are many different health systems or health service providers—who operate in the same territory without coordination, and each provider has its own agenda for delivering healthcare (1, 2). Additionally, fragmentation may occur in settings where public health decisions are made under incomplete information (3). Health system fragmentation is inefficient and contributes to fewer health resources to the people in need (4). Many health systems in low- and middle-income countries (LMICs) remain fragmented, creating health system gaps and adversely affecting low-income patients (1). System fragmentation results in extreme health inequities in the community as it affects the poor individuals and households most, which makes it difficult to achieve universal health coverage (UHC) in LMIC countries (1, 5). Fragmentation prevails in global health agendas and between prominent global health actors (6) and even in the strong economies such as the United States, China, and Europe (7, 8).

The fragmented health system creates pressure on the poorest communities to find easy access to live-saving healthcare services (9). The fragmentation complicates the process of transferring patients from primary or secondary health care services to tertiary hospitals, especially for patients who do not have proper insurance coverage or enough money to cover the treatment or transportation costs (1). Furthermore, health system fragmentation leads to delays in diagnosis and treatment, as patients cannot afford to pay for medical treatment, which aggravates the clinical conditions and eventually lead to complications and late-stage diagnosis, and even more financial burden. As a result, mortalities and morbidities within poorest communities upsurge (1).

Health system fragmentation puts the poorest patients at risk of financial hazards due to soaring out-of-pocket payments (OOP) on the basic healthcare services. As a result, the health care expenses rise and can drive families further into impoverishment (1). Many poor families in LMICs do not have health insurance, and they tend to spend all their savings or sell major assets when a family member gets sick (1, 5). The incompatibility between community expectations for affordable, good health quality, and the fragmented health system increases the pressure on health authorities and politicians (10).

Yemen is considered one of the most impoverished countries in the Middle East with more than half of the population suffering from poverty of various severity and lack access to essential services, including water and health (11). The continuous and protracted conflict in Yemen has evolved into the worst humanitarian situation in modern history, with all public structures, including the health system being put under tremendous pressures. One of these key obstacles to improve the health outcomes in Yemen and to strengthen the health system is the fragmentation of the health system (12). The history of poverty and very long political instability, combined with more than 6 years of war, have destroyed infrastructures, collapsed economy, increased the number of displaced people, and resulted in paralyzed public institutions and inaccessible services in the country (4, 13). Moreover, the conflict destroyed the remaining basic health infrastructure. WHO estimated that almost 50% of the Yemeni public health facilities are not fully functional, and even those fully functional ones are in a dilemma of severe shortages of staff, basic equipment, and essential medicines (14). The fragmented and fragile health information system has been a prominent hurdle to attain informed decision making in the health sector in Yemen. There was a manifestation of irregularities of reports, incompleteness, and incorrectness of data, infrastructure and technology constraints, and weakened human resource capacities to synthesis, analyze, disseminate, and use health data (15–17). Moreover, transparency on how data form health system are collected, analyzed, and used are minimal, sensitive, and challenging (18).

The private sector has been growing over the last two decades in Yemen. However, signs of fragmentation are evident with weak governing and regulatory bodies and policies, inequitable distribution of private facilities between rural and urban areas, arbitrary engagement with the public sector, and issues related to data, quality, and service cost discrepancies across private service providers of different sizes and types such as small clinics, medium polyclinics to large hospitals owned by non-government organizations (19–21).

Studies addressing health system fragmentation during conflict settings with a focus on Yemen are scarce and limited. To our knowledge, there is no study on fragmented health system in Yemen. Therefore, this study explores and documents the different forms of fragmentation in the Yemeni health system.

## Materials and Methods

### Study Design

This study adopted a qualitative research methodology to get an in-depth understanding of participants' perceptions of the fragmentation in the Yemeni health system. This study is part of a larger research effort that used the WHO's General Program of Work−2019–2023 (GPW 13) as an integrative framework to understand the health system fragmentation in Yemen and its forms (22).

### Sampling

The researchers employed purposive sampling to select key health informants for interview. Key informants were selected for in-depth interviews from different organizations, including the Ministry of Public Health and Population (MoPHP) and international and local non-governmental organizations working in Yemen.

The researchers used a semi-structured interview guide, asking about the forms of fragmentation and synergies in the Yemeni health system in relation to the implementation of the main strategies, Universal Health Coverage (UHC), Health Security (HS), and Health Promotion (HP).

Purposively, we invited 20 key informants via email to participate in the study and to secure the representation of different organizations and roles. The invitation was based on their affiliations and their relevant experience in the health system and health policies in Yemen. The invited 20 key informants were from the following institutions: MoPHP (six), International Organizations (six), independent consultants (four), and Academia (four). Six invitees refused to participate, and another six did not reply. Eight invitees have confirmed their participation and, therefore, composing the final sample size for the interviews. Among the eight participants, three of them are UN staff, two are from the Yemeni-MoPHP (from both north and south), and the remaining three are independent—two Yemeni health system consultants and one academic expert. The key informants represented different stakeholders in Yemen, and no one had a political affiliation to any of the conflicted parties to ensure the neutrality of the study.

Interviews were pre-scheduled at participants' convenience and took place online. We conducted the interviews in Arabic and English languages via phone or video call. In average, each interview took 40 min. We used an in-depth interview guide and took notes during interviews. These audio recordings and notes were securely saved using a special secure number to ensure participants' anonymity and confidentiality.

### Data Analysis

After completing all the eight interviews, all audio recordings were transcribed by the investigator. Data saturation was reached at the end of the eighth interview, when no new information was discovered in the analysis.

Then two researchers analyzed the interview transcripts independently using manual qualitative content analysis (23). Four steps were conducted to gain insight into the key informants' perception of health system fragmentation. First, the interpretation of the data was started by reading each interview transcripts and underlining significant statements. Second, all underlined statements were coded across each interview undergoing inductive analysis. Third, all codes were grouped into two themes: positive perceptions and negative perceptions; however, we presented the fragmentations (negative perceptions) in this manuscript. Finally, all researchers re-read all statements in both themes to reflect on the overarching respondents' perception of health system fragmentation. Collaborative discussion with the research team about the themes and findings was conducted to ensure the reflexivity of researchers' experiences for consensus building, reliability, and data saturation.

## Results

**Figure 1:**
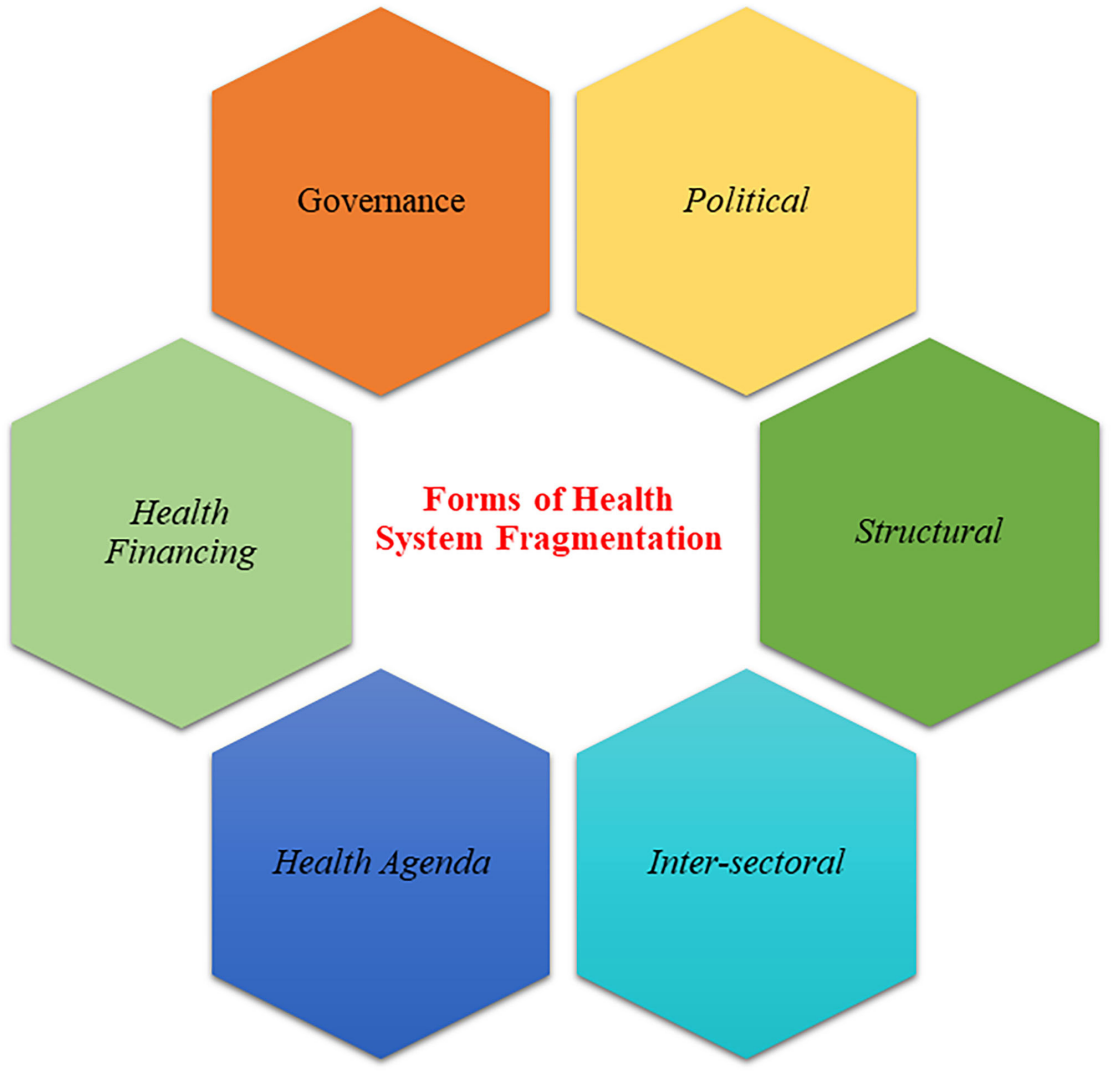
Health system fragmentation forms in Yemen.

Based on the themes presented in the literature and described by our interviewees, we drew out six fragmentation forms. These fragmentation forms reflect the current fragile situation in Yemen particularly for health system. The six sets of fragmentation forms are political, governance, health financing, structural, health agenda, and intersectoral fragmentation (Figure 1).

### Political Fragmentation

Political fragmentation is a result of the current conflict and the presence of different parties and different political interests. All key informants mentioned that the presence of two governments in the country (one in the south and another one in the north) is one of the critical challenges that block the effective implementation of health programs and projects in Yemen. The end result is that, there are two different ministries of health with different leaderships (ministers and technical staff) in the same country.

“Since 2015, *we have war and conflict situation which is really difficult situation to implement the development policies that we have at MoPHP, so […] fragmentation can occur at the high level of decision making. Sometimes you can find at the MoPHP, the departments are not connected together to have horizontal implementation of activities”* **Informant # 1 (Public Health Expert)**“*The fragmentation is clear, because if we are talking about the terrains under the International Recognized Government and the terrains under Ansar Allah, they have their own ministers, they have their own systems. Even some agencies and donors are trying to push to have one project for all country areas; at the end, you will find the rejection from both International Recognize Government and the de facto authorities. Therefore, each party controlling an area would like to have its own voice and its own decisions. This situation also has affected the work of the humanitarian organizations and affected the people health”* **Informant # 7 (UN staff)**.

### Governance Fragmentation

This form of fragmentation is due to the lack of effective leadership at central level of the health system. Weak governance from both ministries of health exists; however, at the governorates' level, the situation is slightly better with clearer guiding role of health offices. One of the participants explained the weak governance and the influence of donor on health sector by stating:

“*The donors who give money always control, you know, the people who have money always control! Therefore, when they give money for local partners, […] the local partners adapted their selves based on the interest of the donors. […], the governance is really weak, they cannot control these local partners or national partners, so the money is controlling them”* **Informant # 1 (Public Health Expert)**.“*You know if we have weak governance and weak leadership, we cannot really control these donors and international actors and even national actors all those people are working abroad, I mean the MoPHP should be the umbrella for all those players, but now especially with the current conflict, we found the health cluster is the umbrella. The health cluster is led by WHO, and we found the role of MoPHP in controlling or covering these activities is weak, the MoPHP should be the umbrella for the health cluster not as part of the cluster like any partner, so this is because of the weakness in the governance*” **Informant # 1 (Public Health Expert)**.

Another cause of this fragmentation captured by the same interviewee was the weak communication process and suboptimal coordination between the MoPHP and international partners. This often leads to duplication of work and lack of streamlining key priorities, resulting in expensive and inefficient interventions in some cases. As one of our interviewees summed up:

“*There should be a more engaging mechanism to involve the MoPHP in the leadership of the health cluster, even thou we are suffering from fragmentation within health system itself by having two ministries, one in the north and another in the south”* **Informant #2 (Public Health Expert)**.“*for example, if you read the list of the minimal health service package there is no preparedness for outbreaks, the focus on communicable disease only on Malaria and TB, and this is another fragmentation because they focus only on two diseases which are supported by the global fund, so this is a type of fragmentation within these good initiatives from the international or humanitarian community*” **Informant #1 (Public Health Expert)**.

Another interviewee elaborated that the weak fragmented leadership is not only a result of the ongoing conflict but was also there even before the war. The same interviewee confirms that the MoPHP demonstrated weak governance as can be evident by its inability to guide building health facilities:

“*For me, fragmentation exists before the war [….], we discovered during HeRAMS, that there were health facilities established of which the MoPHP in Sana'a does not know, it's not in their plans, because it was established by the governorates and by districts*” **Informant # 3 (UN Expert)**.

Another interviewee observed that health sector has been recently led by politicians more than technicians:

“*first of all, the leaders in the MoPHP now are new faces, most of them are politicians not technical people, so there is no priority for the technical perspective during decision making. In addition to the issue of migration of the technical and experienced people from Yemen”* **Informant # 4 (Academia-Public Health)**.

### Health Financing Fragmentation

An interviewee stated that the actors allocate funds mainly for vertical programs, and they ignore the strengthening of the entire health system, and the coordination between the key donors and the MoPHP plays an important role in distributing funds equally, to serve health system strengthening and to prevent fragmentation.

“*Most of the funds coming to the MoPHP are from the international organizations, so it does not come as integrated, it's really fragmented and strengthen the fragmentation at the national level, for example, we will find the global fund, is focusing on the health security and support malaria, TB and HIV, this is mainly going to these three vertical programs, and I do not see fund from the global fund goes for strengthening the health system as a whole”* **Informant # 1 (Public Health Expert)**.

All key informants appreciate the payment of incentives by the international organizations to health officials and health workers; however, there are some negative issues mentioned related to the mechanism and amount of incentives that affect the current health system:

*One of the points that were put forward today is giving the incentives through the MoPHP, but giving a certain amount for six months and then stop it, causes problems with the health workers. The incentives should not exceed the actual salary, for example, if his salary 80,000, the incentive must be around this amount or similar, but to give high amounts, for example, $ 300 this is causing problems for us”* **Informant # 8 (MOPHP)**.“*The disbursement of incentives and per dium in a very large amount, that led to the devastation of the health system and the migration of people from government health facilities to the private sector and international organizations. In addition to problems in the health facility, not all health workers within the health facility receive incentives therefore all work on concentrating on the people who receive the incentives, if these incentives stopped, so the paid workers did not work, the first group stopped because they did not get an incentive from the beginning and the second group stopped because the incentive did not continue, so they ruined the whole health system.”* **Informant # 4 (Academia-Public Health)**.

### Structural Fragmentation

This form of fragmentation is demonstrated within the MoPHP sectors and program structure. Interviewees suggest that there is a lack of integrated model for health service provision and management. They argue that there is insufficient collaboration and coordination between different health sectors and programs within the MoPHP at national level. Interviewees explain:

“*Sometimes you can find at the MoPHP the departments are not aliened together to have horizontal implementation of activities, however at the facility level or at community level you can find all this agendas are there together in the same place, but when you go to the upper level there is many issues you can see, because of the fragmentation of decision at the higher level”* **Informant # 1 (Public Health Expert)**.“*We want an advocacy for health at MoPHP level and among its workers. Unfortunately, within the Ministry each sector [….] is independent and separated, the ministry is inconsistent with its sectors”* **Informant # 4 (Academia-Public Health)**.

### Agenda Fragmentation

Agenda fragmentation is related to the national health agenda. Responders confirmed that the National Health Strategy (NHS) 2010–2025 is the main strategy at the national level that focuses on the access to health services and HS, but they ignore the HP part. However, lack of knowledge of NHS at the peripheral level can cause fragmentation during the implementation.

“*Perhaps I participated in the development of the National Health Strategy before the war, at that time we focused on the principle of the PHC health for all, we were not familiar with the term UHC. The UHC principle came with the Sustainable Development Goals in 2015 after the development of this strategy. Furthermore, there was no advocacy for this strategy at the subnational level to develop micro-plans based on this strategy and use it at governorate and district levels, who will implement these strategies? Of course, the peripheral level. How they can reflect the strategy in their implementation if they do not know about it”* **Informant # 4 (Academia-Public Health)**.

Two interviewees pointed the influence of international organizations and their activities on national health policies and agendas. They argue that uncoordinated actions from different global actors had negative impact on the health system leading to more fragmentation and deterring achievement of UHC. They explain:

“*For your information, part of the fragmentation in the health system caused by the international Organizations, they are implementing policies, strategies, and agendas that did not serve the health system. On the contrary, such things lead to the destruction of the national health system, for example, the implementation of MSP in the current way is destroying what is broken”* **Informant # 4 (Academia-Public Health)**.“*WHO and other agencies contributed to have this fragmentation by isolating the health security from the overall UHC efforts. They are leading this part and leaving the other part more or less to the MoPHP and other international organizations. So yes, when it comes to UHC and health security, I think there is fragmentation, and the reason behind this fragmentation is the current practice that is being implemented by international donors”* **Informant # 2 (Public Health Expert)**.

### Inter-sectoral Fragmentation

The inter-sectoral collaboration between MoPHP and other sectors in Yemen is one of the fragmentation forms. Most of the interviewees referred to the role of different ministries in fragmenting the health system. Some ministries such as the Ministry of Defense (MoD) and Ministry of Interior (MoI) are providing health services for their staff without coordination with MoPHP through their own health facilities and health staff.

“*The MoD and MoI are providing health services, but they will never include it within the setting of any agenda of national vision or strategy […], let me tell you why, they will not be involved because usually their services are provided to their staff (Cadre), which could be the army or could be the police, in this case”* **Informant # 6 (MoPHP)**.“*There is no role for the MOD, the MOI or the Ministry of Finance, to what happens in the MoPHP, everyone works alone and separately. The opposite is true, that the MoPHP are supporting programs in the MoD and the MoI, especially in the north”***Informant # 5 (Formal UN Staff)**.

Another interviewee indicated the absence of integration in fighting malnutrition in Yemen by saying:

“*There is no integration in all services that we are providing to the children, if we are starting good with Severe Acute Malnutrition (SAM) cases going to Moderate Acute Malnutrition (MAM), and we stop the services for MAM cases, he or she will go back to be a SAM case and to die if there is no further support [….], to be treated and after one week without having any food at home so definitely will go back to be severe case, so this kind of situation needs integration between all sectors”* **Informant # 7 (UN Staff)**.

Another interviewee explains how response to cholera outbreak in Yemen requires well-established coordination and collaboration mechanisms between different sectors:

“*The solution for the cholera control is by working in different teams with the collaboration of the MoPHP and other sectors like water and sanitation, cleaning Fund, local authorities, Ministry of Public Works and Ministry of Electricity, everyone working in this direction with the partnership with UNICEF and WHO, and especially UNICEF which is engaged in WASH”* **Informant # 8 (MoPHP)**.

## Discussion

Our analysis shows that there are several forms of fragmentation of the health system in Yemen. These forms of fragmentation have hindered the implementation of national strategies, as well as global health agendas such as Universal Health Coverage, Global Health Security, and Health Promotion. Fragmentation erodes the effectiveness of health programs and jeopardizes the attainment of the Sustainable Development Goals (SDG 3). Globally, the consensus around minimizing health system fragmentation is growing in order to achieve UHC (2).

While authors' conceptualization of fragmentation in this research is seen as a factor explaining many ills of the health system, it is important to realize that fragmentation is a result of several factors. Health system is a reflection of the government system and state rules and actions. Underlying conditions such as violence, impoverishment, under-funding, misrule, and poor central government, which have afflicted Yemen since its birth as a state, most likely have resulted in this state of health system fragmentation and probably in other sectors.

Yemen has entered an era of political turmoil since the begging of 2011, and then later in March 2015, the whole country arrived at the current war. Political fragmentation is a prominent result of the current conflict in the country and the presence of different parties with variable political interests. Political fragmentation is one of the most prominent forms. In Yemen, different political parties have different interests and levels of power. The presence of two distinct régimes and ideologies in the country has created different health policies and strategies being implemented within the same country. The political fragmentation in countries has undermined the decision-making process at a high level that also affected the implementation of the global health agenda and integrated health programing (22). This is evident in the Yemeni context, with the existence of two separate entities for the MoPHP, which, in turn, broadens the scope of the fragmentation between the northern and southern halves of the country. Ensuring successful programing necessitates that key health actors and stakeholders pay careful attention to these divisions and differences when implementing programs and projects and understanding the local dynamics, which is inconvenient. The political interests and affiliation of decisions makers have been reflected into the health system at national and subnational levels within the health institutions as well, even in the same one-party territorial influence (of either the recognized government or Ansar Allah).

Governance fragmentation reflects weak leadership and governance in both MoPHPs in the north and in the south of the country. Literature confirms long-lasting challenging issues related to health system governance in Yemen (24). Limited evidence-based decision making is widely observed and documented by the literature and interviews (24, 25). Fragmented health information system is clearly linked to weakened governance, limited and poor evidence-informed policy, and decision making in the health sector (17, 26). Data analysis, and use and sharing in Yemen are even more cumbersome during humanitarian and crisis times leading to limited empirical evidence generation and usage (26). The weakness of leadership and governance of the health system in Yemen during the protracted conflict has paved the way for further fragmentation. In light of the scarcity of financial resources available to the MoPHP and depending on the international organizations, the leadership has shifted to these organizations to direct and define the health agenda priorities in the country. Therefore, it has been observed that many local actors in the health sector have adapted to the requirements of supporters and implemented specific silo programs and institutional agendas that serve specific mandates. Weak coordination and communication between health stakeholders themselves, on one hand, and with MoPHP, on the other hand, support possibly more fragmentation. Hence, harmonization and alignment within the health sector have been more challenging. Unsurprisingly, bringing MoPHP back to the driving seat was neither a priority nor an objective for any major actor in Yemen. Likewise, it was documented from several fragile and post-conflict states (27).

Fragmentation of health financing has been documented in some countries (6). Financing health programs over the years of conflict has been extremely donor dependent and firmly linked to high private out-of-pocket spending. International donors' funding to health was characterized as fragmented even before the humanitarian era in the country mainly due to the vertical program approach rather than system-wide strengthening support (4). Since conflict eruption in early 2015, donors choose to channel health funds exclusively through non-governmental organizations (NGOs) more willingly than local health authorities do. Nevertheless, it is widely recognized that the NGOs cannot fulfill the role of the national authorities, and such approach might be a weakening factor to MoPHP roles as a steering body for the health system (28). Allocation of resources in the health sector is not often equitable. In many cases, they are distributed based on the donor's interest, which often aims to reduce risks from a specific disease, and, in fewer cases, based on the recipient's interest (29).

Structural fragmentation is another form that emerged from this analysis mainly within the MoPHP in Yemen. Integrated and comprehensive health services that are well-coordinated by the MoPHP are fundamental to minimize health system fragmentations. Key stakeholders, with the WHO in the lead, worked with health authorities to review the service package in 2016 and arrived at the minimum service package (MSP). The goal behind MSP was to ensure and increase access to health services during crises in scarce-resource settings and to strengthen the fragile health care system by improving the quality of the basic health services. As part of MSP support, to maintain service provision by paying health workers monthly incentives, health workers, due to fund shortages, did not receive their salaries for a long period (30). Many respondents believe that the implementation of the MSP does not serve the health system and even it might participate in enduring the health system. The performance-based incentives are recognized approaches to maintain health services and guarantee conditionally its sustainability, yet high incentives (more than staff salary scales) have an undesirable impact on the health system (31, 32). MSP could be an opportunity to minimize fragmentation if MoPHP uptake is ensured and supported to steer the implementation of this package countrywide. In Yemen, health system continues to have many actors with different agendas. The current humanitarian situation has led to the proliferation of NGO actors in the country including health actors. In reality, fragmentation between multiple actors' priorities and agendas due to weak coordination is inevitable. Literature documented that health sector stakeholders and institutions have their own mandates and prioritize their own agendas, lacking coordination with others, which may negatively affect the implementation of health programs or sector-wide strategies (33, 34). Health security has been in isolation from the UHC, and health promotion is usually overridden by many stakeholders; thus, health programs and projects are driven mainly by international donors and organizations rather than the MoPHP.

Sectoral coordination and collaboration beyond MoPHP and the health system are suboptimal in Yemen (25). Inter-sectoral fragmentation jeopardizes MoPHP efforts to achieve wider health gains and health system resilience alike in times of crisis. The existence of two ministries in the same country, and sometimes in the same governorate, has increased health system fragmentation. Prominent weak integration between MoPHP and other sectors such as water and sanitation, public work funds, local authorities, and ministries of defense and interior in Yemen is most probably due to lack of knowledge about the importance of prioritizing health in all other policies in the country (25, 35). Integration is instrumental for poverty reduction and outbreak containment, through improving access to basic and life-saving health and social services. Additionally, integration between the private and public sector is of limited scope with a handful of pitfalls (17, 19). This includes fragmentation in service provision across different geographical areas, data sharing, and management, regulation, and policy making.

Although health system fragmentation is predominantly observed in many different forms in Yemen, windows of opportunities exist. Despite the challenges indicated by interviewees related to MSP implementation, it has a potential to be a base for further integration of health services, in particular, at the primary healthcare level and, thus, contributing to UHC. Lessons from Yemeni context were drawn on challenging integration such as in response to cholera outbreak (36). Yet, the recurrence of several outbreaks over a few years demonstrates the fragility and impuissance of the health system even with efforts to foster intersectoral collaboration. MoPHP has a national framework for health service integration at the primary level (37), yet to what extent this guiding strategy has been informing humanitarian interventions or the MSP implementation remains questionable. For example, integrated outreach activities have been irregular and intermittent, which might adversely affect the aims to reach the prioritized population and UHC.

While the study provides substantial evidence of health system fragmentation, several limitations were recognized. The limitations, in one way or another, may have affected the data results. The current conflict limited the researchers to conduct a field visit to the sites; thus, internet connection becomes a problem. The current war and conflict in Yemen made the data collection process difficult. Several key actors in the MoPHP refused to participate due to security fears and political chaos. Furthermore, the existence of two health ministries in the country makes the situation even worst. The participants may be scared to express what they really felt, which may dilute the information gathered. This information bias was reduced through constant reassurances of the participants that only the investigator will handle the interview transcripts. Moreover, it was emphasized that their names and their affiliation will not be in any documents, and that numbers were given to provide confidentiality and anonymity.

This study does not include all forms and contributed factors of the fragmentations in Yemen, such as fragmented health information, and further studies may explore more forms of fragmentations. Finally, there may be a personal bias in the interpretation of the qualitative data by the investigators. The study identifies six sets of health system fragmentation forms in Yemen during the protracted conflict and complex humanitarian situation, which are political, governance, financial, structural, agenda, and intersectoral fragmentations. The protracted conflict and fragile situation in Yemen have accentuated the health system fragmentation. Better understanding of these forms of health system fragmentations and prioritization of the health system approach can minimize fragmentation, improve effective programing, and contribute to UHC in Yemen. The researchers call all actors in the health sector to specifically address these forms in their programs and interventions, and build consensus on health system agenda. Health system analysis in Yemen can be very beneficial to build a common ground and priorities to reduce health system fragmentation and to support the humanitarian development nexus.

Despite the importance of intersectoral coordination and the existence of humanitarian work standards represented by SPHERE, this study documents health system fragmentation by the identified six forms. Authors recommend further in-depth exploration of the drivers for each form of fragmentation. One major driver could be the urgency and pressure on key stakeholders to take quick actions in light of emergency situations in the interest of saving lives, for example, regardless of the process in between which might be time consuming. Therefore, programming efficiency and efficacy might, in turn, be affected or overlooked.

National and local health authorities have major influence on the success of humanitarian actions, and their role in facilitating the implementation at the ground is mountable. Henceforth, their capacity building and its prioritization is fundamental to positively bring about more integration and gains in health situation and response. Likewise, health sector capacity building is a necessary investment for the humanitarian development nexus and the recovery efforts in the future when peace hopefully returns sooner than later to Yemen.

## Data Availability Statement

The raw data supporting the conclusions of this article will be made available by the authors as per request. further inquiries can be directed to the corresponding author/s.

## Ethics Statement

The study adheres to Declaration of Helsinki on ethical clearance, informed consent, ensure the privacy, confidentiality of the research participants, and also covering the risk and the benefits of the research to the participants (38). The researchers secured Ethical clearance from the Ethical Committee of the Ruprecht-Karls-Universität Heidelberg (S-182/2019). Moreover, Individual verbal and written informed consents were acquired from all participants prior to their participation.

## Author Contributions

FD, TH, RS, NA-D, SA-A, and AJ have been sufficiently involved in this submission to take public responsibility for the work, meaning that each author has made substantial contributions to the conception and design of the study. FD and TH acquired, analyzed, and interpreted the data. FD, RS, NA-D, SA-A, and AJ drafted the article and revised it critically for important intellectual content. All authors contributed to the article and approved the submitted version.

## Conflict of Interest

The authors declare that the research was conducted in the absence of any commercial or financial relationships that could be construed as a potential conflict of interest. The handling editor declared a past co-authorship with two of the authors FD and AJ.
